# Systems toxicology identifies mechanistic impacts of 2-amino-4,6-dinitrotoluene (2A-DNT) exposure in Northern Bobwhite

**DOI:** 10.1186/s12864-015-1798-4

**Published:** 2015-08-07

**Authors:** Kurt A. Gust, Bindu Nanduri, Arun Rawat, Mitchell S. Wilbanks, Choo Yaw Ang, David R. Johnson, Ken Pendarvis, Xianfeng Chen, Michael J. Quinn, Mark S. Johnson, Shane C. Burgess, Edward J. Perkins

**Affiliations:** Environmental Laboratory, US Army Engineer Research and Development Center, EL-EP-P, 3909 Halls Ferry Rd, Vicksburg, MS 39180 USA; Institute for Digital Biology, Mississippi State University, Starkville, MS 39762 USA; Translational Genomics Research Institute, Phoenix, AZ 85004, USA; Badger Technical Services, San Antonio, TX 71286 USA; Conestoga-Rovers & Associates, Dallas, TX 75234 USA; University of Arizona, School of Animal and Comparative Biomedical Sciences, Tucson, AZ 85721 USA; Bio5 Institute, University of Arizona, Tucson, AZ 85721 USA; IFXworks LLC, 2915 Columbia Pike, Arlington, VA 22204 USA; US Army Public Health Command, Aberdeen Proving Ground, Aberdeen, MD 21010 USA; University of Arizona, College of Agriculture and Life Sciences, Tucson, AZ 85721 USA

**Keywords:** Transcriptomics, Proteomics, Systems toxicology, Nitrotoluenes, Northern Bobwhite, PPAR signaling

## Abstract

**Background:**

A systems toxicology investigation comparing and integrating transcriptomic and proteomic results was conducted to develop holistic effects characterizations for the wildlife bird model, Northern bobwhite (*Colinus virginianus*) dosed with the explosives degradation product 2-amino-4,6-dinitrotoluene (2A-DNT). A subchronic 60d toxicology bioassay was leveraged where both sexes were dosed via daily gavage with 0, 3, 14, or 30 mg/kg-d 2A-DNT. Effects on global transcript expression were investigated in liver and kidney tissue using custom microarrays for *C. virginianus* in both sexes at all doses, while effects on proteome expression were investigated in liver for both sexes and kidney in males, at 30 mg/kg-d.

**Results:**

As expected, transcript expression was not directly indicative of protein expression in response to 2A-DNT. However, a high degree of correspondence was observed among gene and protein expression when investigating higher-order functional responses including statistically enriched gene networks and canonical pathways, especially when connected to toxicological outcomes of 2A-DNT exposure. Analysis of networks statistically enriched for both transcripts and proteins demonstrated common responses including inhibition of programmed cell death and arrest of cell cycle in liver tissues at 2A-DNT doses that caused liver necrosis and death in females. Additionally, both transcript and protein expression in liver tissue was indicative of induced phase I and II xenobiotic metabolism potentially as a mechanism to detoxify and excrete 2A-DNT. Nuclear signaling assays, transcript expression and protein expression each implicated peroxisome proliferator-activated receptor (PPAR) nuclear signaling as a primary molecular target in the 2A-DNT exposure with significant downstream enrichment of PPAR-regulated pathways including lipid metabolic pathways and gluconeogenesis suggesting impaired bioenergetic potential.

**Conclusion:**

Although the differential expression of transcripts and proteins was largely unique, the consensus of functional pathways and gene networks enriched among transcriptomic and proteomic datasets provided the identification of many critical metabolic functions underlying 2A-DNT toxicity as well as impaired PPAR signaling, a key molecular initiating event known to be affected in di- and trinitrotoluene exposures.

**Electronic supplementary material:**

The online version of this article (doi:10.1186/s12864-015-1798-4) contains supplementary material, which is available to authorized users.

## Background

2,4,6-trinitrotoluene (TNT) has been used as a munitions constituent (MC) for over a century and represents a critical environmental contaminant found in soils at ammunition production facilities as well as on military training ranges where ordinance are fired and detonated [1]. Although the toxicity of the TNT has been broadly characterized [2], the effects of key environmental degradation and biotransformation products are still not well understood. 2-amino-4,6-dinitrotoluene (2A-DNT) has been identified as a principle environmental breakdown product resulting from anerobic biodegradation of TNT [3] and remains a chemical of concern for the risk assessment of MC-contaminated sites [4].

Wildlife bird species represent important potential receptors of MCs, especially ground foraging birds that may inadvertently consume these chemicals when ingesting grit or soils to assist with digestion [5]. Quinn et al. [6] examined the toxicological effects of 2A-DNT in the ground foraging bird species, Northern bobwhite (*Colinus virginianus*), and found that it was acutely toxic only at high doses (LD_50_ = 1167 mg/kg). However, in subchronic 60d exposures, sustained oral dosing of 2A-DNT was observed to elicit mortality at a much lower dose (14 mg/kg-d, [6]). In addition to mortality, 2A-DNT dosing caused a variety of sublethal effects in the subchronic exposures including: increased liver weights, decreased leukocyte counts, increased plasma-triglyceride levels and splenic reticular cell hyperplasia [6]. Northern bobwhite exhibited similar effects when exposed to other nitrotoluenes including 4A-DNT [7] and 2,6-DNT [8, 9], however 2,6-DNT tended to have a broader range of sublethal impacts relative to 2A-DNT and 4A-DNT.

Given its attributes as an important avian ecotoxicological model, genomics characterization and gene expression tools have been generated for Northern bobwhite [10–12] providing resources for in-depth systems toxicological assessments. The transcriptomics tools developed for Northern bobwhite have provided mechanistic insights into the toxicology of the nitrotoluene 2,6-DNT providing hypothetical mechanisms of action for a variety of toxicological phenotypes [11]. A principle finding was enrichment and apparent antagonism of peroxisome proliferator-activated receptor (PPAR) signaling pathways that were hypothesized to result in impaired energy metabolism and ultimately lethargy and weight loss [6, 11]. Similar responses were observed in Sprague–Dawley rats for multiple nitrotoluenes in addition to impaired lipid metabolism [13], a principle metabolic pathway regulated by PPAR signaling [14]. Recently, Wilbanks et al. [15] conducted experiments with PPARα knockout mice combined with *in vitro* PPAR nuclear signaling assays validating that antagonism of PPARα by the nitrotoluene 2,4-DNT represents the molecular initiating event (MIE) for impaired exercise performance and weight loss. Overall, these results suggest conservation of this MIE across species and potentially across nitrotoluenes.

In the present study, liver and kidney tissues derived from the subchronic 60d assay described in Quinn et al. [6] were investigated to identify the molecular mechanisms underlying toxicological phenotypes using a systems toxicology approach to compare, contrast and integrate global transcriptomic and proteomic responses to 2A-DNT dosing. Specifically, statistical enrichment of gene networks [16, 17] and canonical metabolic pathways [18, 19] were utilized to identify hypothetical molecular initiating events (MIEs) and metabolic pathway impairment providing key information within adverse outcome pathways [20] for nitrotoluene compounds. Finally, we conducted *in vitro* PPAR nuclear activation/inhibition assays to test if 2A-DNT interferes with PPAR signaling.

## Methods

We utilized tissues from a study by Quinn et al. [6] that characterized the apical toxicological impacts of 2A-DNT in Northern bobwhite. The specific rationale for dose-selections as well as all detailed toxicological results for the 2A-DNT exposures in Northern bobwhite can be found in Quinn et al. [6] Briefly, twelve individuals of each sex were exposed to 0, 0.5, 3, 14, or 30 mg/kg-d 2A-DNT via daily gavage in subchronic 60d bioassays. Immediately following euthanasia by CO_2_ asphyxiation, liver and kidney tissues were collected from each sex and a portion of each tissue was flash frozen for proteomics analyses and another portion fixed in RNA Later™ (Qiagen Inc., Valencia, CA) following manufacturer’s recommendations for transcriptomics analyses. Tissues were stored at −80 °C until needed for analysis. All animal exposure protocols were conducted consistent with Good Laboratory Practices, conducted at the US Army Public Health Command (USAPHC) AAALAC accredited facility and were approved by the Institutional Animal Care and Use Committee at the USAPHC.

### RNA extraction

RNA extraction was conducted as described in Gust et al. [10] and Rawat et al. [11]. Briefly, RNA extraction was conducted using RNeasy Mini RNA extraction kits (Qiagen Inc.). RNA quality was assessed using an Agilent 2100 Bioanalyzer (Agilent Technologies, Waldbronn, Germany) with RNA 6000 Nano LabChips® and a NanoDrop ND-1000 Spectrophotometer (NanoDrop technologies, Wilmington, DE, USA). Only samples with a 28 s/18 s ratio ≥2.0 and an RNA integrity number ≥7.0 were used for downstream applications.

### Microarray experimental design, hybridizations and data extraction

We utilized the 8x15K custom oligonucleotide microarray platform (Agilent Technologies, Santa Clara, CA) developed for Northern bobwhite and described in Rawat et al. [11] for all transcript expression investigations. Microarray hybridizations were conducted using completely randomized design experiments including a 2 × 4 factorial treatment arrangement to investigate the following conditions: sex (male and female) and 2A-DNT dose (control, 3, 14, and 30 mg/kg-d) for both liver and kidney tissues. All conditions included 4 biological replicates. The Agilent One-Color Microarray Hybridization protocol (Agilent Technologies) was utilized for microarray hybridizations following manufacturer’s recommendations. One μg of total RNA was utilized for all hybridizations. An Axon GenePix® 4000B Microarray Scanner (MDS Analytical Technologies Inc., Toronto, Canada) was used to scan microarrays at 5 μm resolution. Data were extracted from microarray images using Agilent Feature Extraction software (Agilent Technologies). Analysis of internal control spikes added prior to cRNA synthesis indicated that signal data was within the linear range of detection. All microarray data and results have been archived at the Gene Expression Omnibus (GSE59910, http://www.ncbi.nlm.nih.gov/geo/query/acc.cgi?acc = GSE59910).

### Microarray data analysis

Background subtracted, adjusted median signal intensities were normalized on a per-chip basis using custom scripts with R (http://www.r-project.org/) software [21]. The script transforms the signal intensity by dividing signal intensity for all the genes with the mean intensity in each array. Microarray analysis was performed using HDArray from Bioconductor (www.bioconductor.org) which utilizes a Bayesian probabilistic framework-based *t*-test to test for differences in gene expression [22]. The normalized data was imported into HDArray and *p*-value associated with fold change was calculated for each gene. The HDArray results output were exported into MySQL (www.mysql.com) and the overlap between log transformed *Bayes p* (*p* < 0.05, unless stated otherwise) and present signal flag for all replicates was taken. The microarray for Northern bobwhite includes 8,454 non-redundant sequences with positive-frame orientations in addition to 3,272 sense-antisense probe-pairs representing transcripts for which the frame-orientation was not known. For the sense-antisense probe pairs, one probe represents a nonsense sequence and our expectation was that no target should have specific binding to it. All probes for which expression was observed in both the forward- and reverse-sequence were considered non-specific and were removed from the expression analysis.

### Reverse-transcriptase, quantitative polymerase chain reaction (RT-qPCR)

Microarray results were validated using RT-qPCR (Additional file 1: Table S1). DNase (Qiagen, Valencia, CA, USA) treated total RNA from all biological replicates previously used in microarray hybridizations (4 replicates per condition) were examined using RT-qPCR (see supplemental text for detailed methods). Briefly, 500 ng of total RNA was reverse transcribed into cDNA in a 6.3 μL reaction containing 250 ng of random primers and SuperScript III reverse transcriptase (Life Technologies, Grand Island, New York), following the manufacturer’s protocol. Cycling parameters for cDNA amplification were 95 °C for 15 min, 40 cycles of 95 °C for 15 s, and 60 °C for 1 min. Results were normalized to 18S rRNA expression and analyzed using the ΔΔCt method (Applied Biosystems, Foster City, CA, USA). The 95 % confidence interval (95 % C.I.) was calculated around the mean relative expression for each treatment. Confidence intervals that did not include unity were considered differentially expressed relative to controls as described in Rawat et al. [11].

### Proteomics experimental design, extractions and sample preparation for mass spectrometry

Protein isolations were carried out using four biological replicates of male kidney, female liver and male liver tissue from birds exposed to either 0 mg/kg-d 2A-DNT (control) or 30 mg/kg-d 2A-DNT (treatment). One hundred mg of each tissue sample was subjected to differential detergent fractionation (DDF) as described by van den Berg et al. [23] and Vergnon et al. [24]. DDF extraction resulted in four protein fractions for each sample: cytosolic, membrane/organelle, nuclear and least soluble. Fifty μg of protein from each DDF fraction was precipitated in 50 % trichloroacetic, washed twice with acetone, and trypsin digested as described by van den Berg et al. [23]. After digestion, residual detergents were removed from the digests using a strong cation exchange macrotrap (Michrom TR1/25108/53, Bruker Corporation, Fremont, CA, USA) followed by desalting using a peptide macrotrap (Michrom TR1/25108/52, Bruker Corporation) according to manufacturer’s instructions. Peptides were dried and re-suspended in 20ul of 5 % acetonitrile (ACN), 0.1 % formic acid for nLC-MS/MS analysis.

### Proteomics - mass spectrometry

Peptide mass spectrometry was achieved using a Surveyor HPLC system (Thermo) configured for nano flow rates using a split solvent supply coupled with an LCQ DECA XP Plus ion trap mass spectrometer (Thermo Thermo Fisher Scientific, Waltham, MA, USA). Peptides were separated with a BioBasic C18 reversed phase column (Thermo 72105–100266, Thermo Fisher Scientific) using an acetonitrile (ACN) gradient of 5 % ACN to 50 % ACN in 620 min. Flow rate was set at 500 nL per minute with 0.1 % formic acid as an ion source. Column eluate was ionized using a stock LCQ nanospray ion source operated at 2 kV applied using liquid junction just before a silica emitter. The LCQ was operated in normal scan mode with MS/MS scans of the top five most abundant ions from each precursor scan. Dynamic exclusion was enabled with a repeat count of two and a duration of two minutes. Data collection occurred over the entire 620 min gradient.

#### Protein identification

Mass spectra were searched against a protein database using the SEQUEST [25] algorithm in Bioworks 3.3 (Thermo Fisher Scientific). At the time of analysis, the *C. virginianus* known proteome contained fewer than 100 proteins so the reference proteome (RefSeq) for *Gallus gallus* containing 18,768 entries was downloaded from NCBI on September 29, 2009. The protein database was *in silico* trypsin digested and cysteine carbamidomethylation and methionine oxidations (single and double) were included in the search criteria. Precursor and fragment ion tolerances were set at 1.5 Daltons. A randomized decoy database was also searched with mass spectra using the same search criteria as described above to estimate the probability of peptide identifications being false positives. We used a peptide probability filter of p ≤ 0.05 for protein identifications. Identified proteins were evaluated for differential expression using Monte Carlo re-sampling statistics [26] at a p-value of ≤ 0.05. The mass spectrometry proteomics data have been deposited to the ProteomeXchange Consortium (http://proteomecentral.proteomexchange.org) via the PRIDE partner repository with the dataset identifier PXD001206.

### Gene network and metabolic pathway analysis

Effects of 2A-DNT exposure on gene network and metabolic pathway inferences were derived using differential expression datasets for transcripts and proteins. Affected gene networks and metabolic pathways were identified using Ingenuity Pathways Analysis software (IPA; Ingenuity Systems, Redwood, CA, USA) and the Database for Annotation, Visualization and Integrated Discovery (DAVID v6.7, http://david.abcc.ncifcrf.gov/, [18]), respectively. Gene lists for were converted to *Gallus gallus* gene homologs for IPA and DAVID analyses. Gene network analysis in IPA calculated p-values for the overlap between the input data and the known molecular interactions curated within IPA knowledgebase. Fisher’s exact tests (p = 0.05) were conducted to determine the probability that each molecular network was enriched by chance. For significantly enriched molecular networks, the top 5 were used to interpret biological outcomes resulting from 2A-DNT exposure. Enrichment of metabolic pathways curated by the Kyoto Encyclopedia of Genes and Genomes Database (KEGG, http://www.genome.jp/kegg/pathway.html) were investigated using DAVID where enrichment was calculated using the *Gallus gallus* reference genome as the background gene set and the cutoff for significant enrichment was *p* < 0.10.

### PPAR nuclear activation assays

To determine the effect of 2A-DNT on PPAR signaling, nuclear receptor reporter assays were conducted (PPARα, PPARγ, PPARδ, and the PPAR co-factor RXRα human cell-based assays, Indigo Biosciences, State College, PA). Cell viability was measured in the nuclear receptor reporter assays using a live cell multiplex assay (Indigo Biosciences). All bioassays were conducted according to the manufacturer’s specifications. Briefly, PPARα, PPARγ, PPARδ, or RXRα − luciferase reporter cells were thawed in cell recovery medium for 10 min at 37 °C. Cells were distributed into 96-well plates, then dosed immediately with test chemicals in compound screening medium at 0.01, 0.1, 1, and 10 mg/l (*n* = 3) diluted from a 2A-DNT stock solution in dimethyl sulfoxide. The final dimethyl sulfoxide concentration in all wells was 0.05 %. Cells were also incubated with their respective agonists as positive controls (PPARα: 100 nm GW590735; PPARγ: 1000 nM rosiglitazone; PPARδ: 30 nM GW0742; RXRα: 1000 nM 9-*cis* retinoic acid, supplied in each assay kit).

After 24 h exposure to 2A-DNT, cells were rinsed with live cell multiplex assay buffer and then incubated with live cell multiplex assay media (containing the acetomethoxy derivate of calcein (calcein-AM)) for 45 min at 37 °C and 5 % CO_2_. Fluorescence of calcein (the cleaved bi-product indicative of cell survival) was measured at 492 nm excitation/513 nm emission with a spectrophotometer (Tecan Safire v2.20, Research Triangle Park, NC). The media containing calcein was discarded and replaced with luciferase detection reagent. Luminescence (relative luminescence units) was measured with a microtiter plate luminometer (Dynex MLX 1000, Dynex Technologies, Chantilly, VA) after a15-min incubation with the luciferase detection reagent. Finally, to determine the antagonistic activity of 2A-DNT, cells were co-incubated with 2A-DNT at 100 mg/l and the respective receptor agonists at the concentrations listed above.

## Results

### Transcript expression

**Fig. 1:**
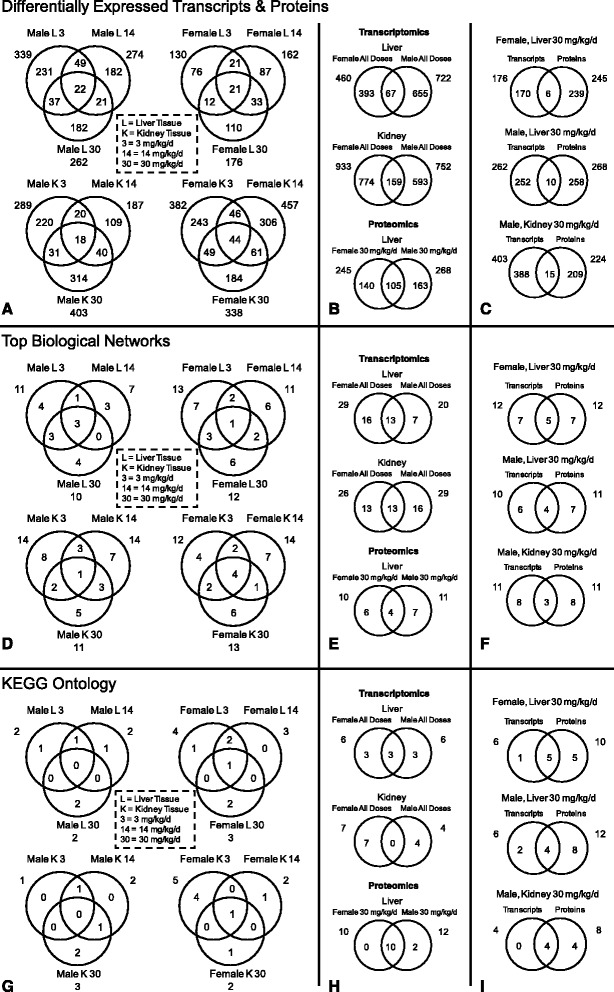
Overlap of: (1) transcripts and/or proteins having significant differential expression (panels **a-c**), (2) significantly enriched biological networks (panels **d-f**), and (3) significantly enriched Kyoto Encyclopedia of Genes and Genomes (KEGG) ontology terms (panels **g-i**) in response to 2A-DNT dosing

As an initial quality control step, a total of 37 and 53 microarray targets for liver and kidney, respectively, were identified to be differentially expressed in both the sense and anti-sense microarray probes and were therefore eliminated from the overall results set. The 2A-DNT exposure elicited significant differential expression of 1,472 transcripts in liver and 2,213 transcripts in kidney tissues across all experimental conditions (Additional file 1: Table S2). Although there were transcripts differentially expressed in common among 2A-DNT dose levels, the overwhelming majority were unique to each dose level (Fig. 1a). The response to 2A-DNT was sex specific (Fig. 1b) where percentage commonality across all doses was ≤ 14.6 % for liver tissue and ≤ 21.1 % for kidney tissue on a transcript-by-transcript basis.

### RT-qPCR results

RT-qPCR provided confirmatory results for the majority of differentially expressed transcripts detected by microarray (Additional file 1: Table S3). Specifically, RT-qPCR confirmed 72 %, 75 %, 68 % and 58 % of statistical test results for microarray in male liver, female liver, male kidney and female kidney, respectively.

### Network analysis (Transcripts)

An overview of the gene network results revealed that, although the commonality of transcriptomic responses across doses and among sexes was limited for transcript-by-transcript comparisons (Fig. 1a and b), 43-67 % of gene networks were common among doses (Fig. 1d) and 45 % of gene networks were common among sexes within both liver and kidney tissues (Fig. 1e).

#### Liver

The most represented IPA gene network functions observed to be significantly enriched in the top 5 networks in liver tissues of males across all 2A-DNT doses included lipid metabolism, cell growth and proliferation, cell death, small molecule biochemistry, and cell development (Table 1). The most highly represented IPA network functions in female liver tissues included lipid metabolism, small molecule biochemistry, cellular development, molecular transport, and cell cycle.

**Table 1:** Top 5 networks observed to be affected in liver tissue based on differentially expressed transcripts or proteins using Ingenuity Pathway Analysis

Transcriptomics results	
Female, Liver, Tissue, 3 mg/k/day	Network function score
Carbohydrate Metabolism, Behavior, Genetic Disorder	21
Cellular Growth and Proliferation, Cell Death, DNA Replication, Recombination, and Repair	19
Cellular Growth and Proliferation, Cellular Movement, Cancer	17
Cellular Growth and Proliferation, Hematological System Development and Function, Inflammatory Response	15
Cell-To-Cell Signaling and Interaction, Hair and Skin Development and Function, Tissue Development	10
Male, Liver Tissue, 3 mg/kg/day	
Antigen Presentation, Cell-To-Cell Signaling and Interaction, Hematological System Development and Function	23
Lipid Metabolism, Molecular Transport, Small Molecular Biochemistry	20
Cellular Development, Cell Cycle, DNA Replication, Recombination, and Repair	15
Lipid Metabolism, Molecular Transport, Small Molecule Biochemistry	15
Cellular Growth and Proliferation, Small Molecular Biochemistry, Cancer	15
Female, Liver Tissue, 14 mg,/kg/day	
Lipid Metabolism, Small Molecular Biochemistry, Cellular Development	61
Cell Death, Cellular, Tissue Development	21
Organismal Injury and Abnormalities, Antigen Presentation, Humoral Immune Response	19
Cancer, Dermatological Diseases and Conditions, Cellular Development	18
Cell Death, Organ Morphology, Lipid Metabolism	15
Male, Liver Tissue, 14 mg/kg/day	
Lipid Metabolism, Small Molecular Biochemistry, Cellular Development	21
Lipid Metabolism, Small Molecular Biochemistry, Cellular Movement	19
Lipid Metabolism, Small Molecular Biochemistry, Vitamin and Mineral Metabolism	18
Lipid Metabolism, Small Molecular Biochemistry, Vitamin and Mineral Metabolism	16
Cell-To- Cell Signaling and Interaction, Tissue Development, Cellular Movement	16
Female, Liver Tissue, 30 mg/kg/day	
Organism Functions, Cell Death, Carbohydrate Metabolisms	19
Lipid Metabolism, Small Molecular Biochemistry, Vitamin and Mineral Metabolism	18
Cellular Growth and Proliferation, Skeletal and Muscular System Development and Function, Cellular Function and Maintenance	16
Lipid Metabolism, Small Molecular Biochemistry, Vitamin and Mineral Metabolism	16
Cellular Movement, Nervous System Development and Function, Organ Development	16
Male, Liver Tissue, 30 mg/kg/day	
DNA Replication, Recombination, and Repair, Protein Degradation, Protein Synthesis	20
Lipid Metabolism, Molecular Transport, Small Molecule Biochemistry	18
Lipid Metabolism, Molecular Transport, Small Molecular Biochemistry	17
Cell Cycle, Cellular Development, Cellular Assembly, and Organization	17
Cell Cycle, Hair and Skin Development and Function, Cellular Development	15
Proteomics Results	
Female, Liver 30 mg/kg/day	Network Functions Score
Cell-To-Cell Signaling and Interaction, Tissue Development, Lipid Metabolism	28
Cell-To-Cell Signaling and Interaction, Tissue Development, Cell Morphology	18
Amino acid Metabolism, Small Molecular Biochemistry, Cellular Assembly and Organization	18
Drug Metabolism, Endocrine System Development and Function, Lipid Metabolism	18
Cell Death, Cellular Growth and Proliferation, Nervous System Development and Function	17
Male, Liver 30 mg/kg/day	
Organismal Injury and Abnormalities, Respiratory Disease, Genetic Disorder	24
Lipid Metabolism, Molecular Transport, Small Molecular Biochemistry	22
Cell Morphology, Cellular Development, Nervous System Development and Function	18
Lipid Metabolism Small, Molecular Biochemistry, Molecular Transport	15
Gene Expression, Endocrine System Development and Function, Nervous System Development and Function	2

#### Kidney

For kidney tissues: cell cycle, cellular growth/proliferation; DNA replication/recombination/repair; cell death; cellular assembly/organization; and small molecule biochemistry were the most represented functions in the top 5 IPA networks in females (Additional file 1: Table S4). Cell-to-cell signaling/interaction, cellular assembly/organization, cellular development, and small molecule biochemistry were the most represented functions in males (Additional file 1: Table S4).

### Canonical pathways (Transcripts)

Significant transcript enrichment was observed for 23 KEGG pathways in both liver and kidney in response to 2A-DNT across all doses and sexes (Additional file 1: Table S5). Using apical-level KEGG ontology (KO) terms, commonality of KO terms across doses ranged from 0-100 % (Fig. 1g) and 50 % and 0 % of KO terms were common among sexes for liver and kidney tissues, respectively (Fig. 1h).

#### Liver

**Fig. 2:**
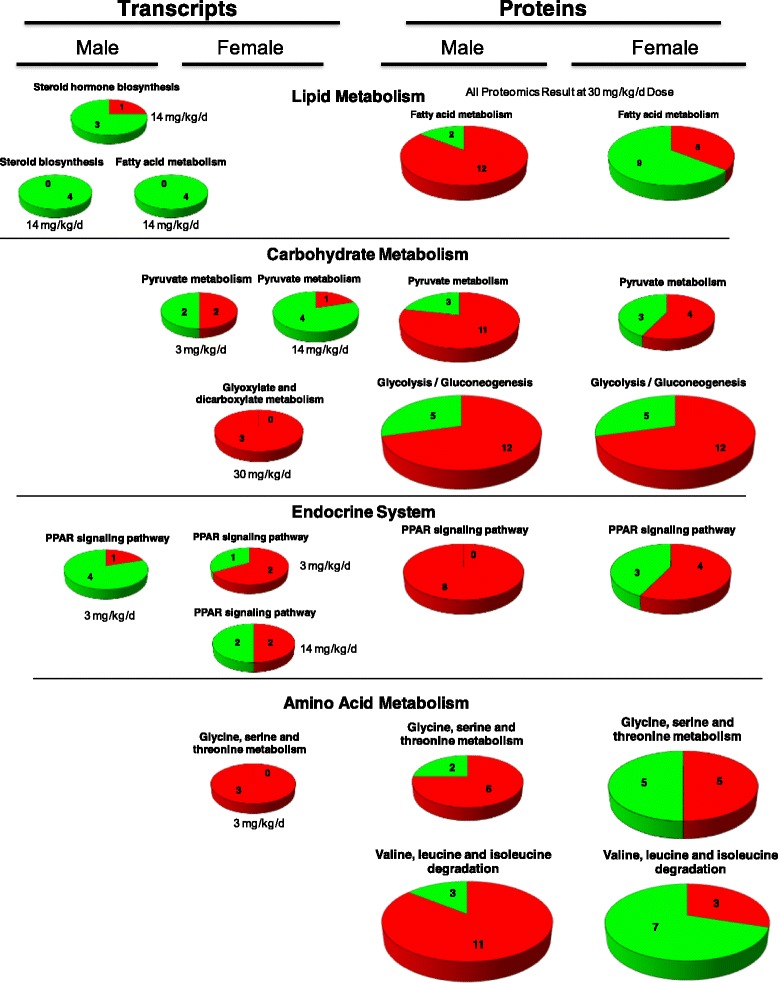
Expression profiles of the most highly enriched Kyoto Encyclopedia of Genes and Genomes (KEGG) pathways found in common among transcriptomic and proteomic results sets representing effects of oral 2A-DNT dosing in liver tissue

**Fig. 3:**
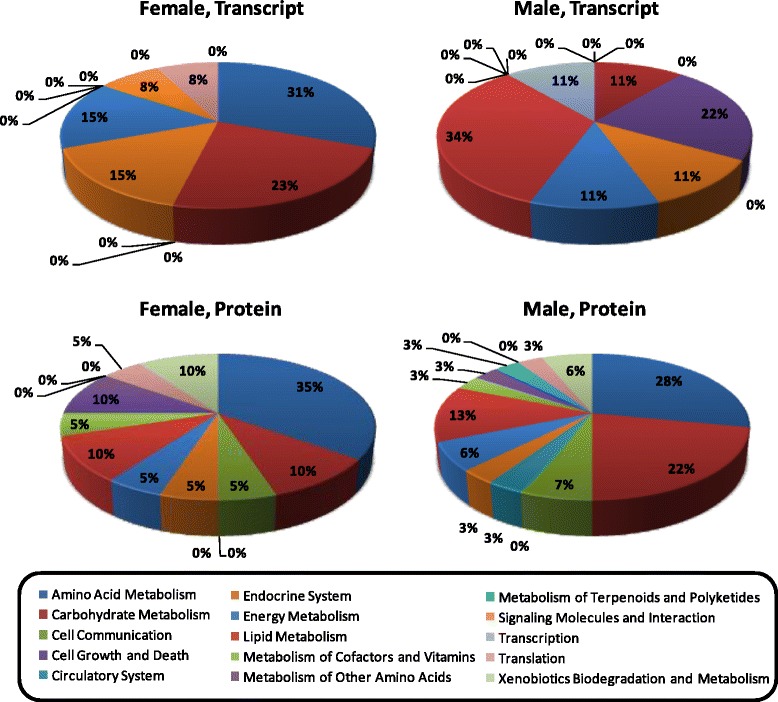
Overview of Kyoto Encyclopedia of Genes and Genomes (KEGG) metabolic pathways enriched in liver tissue of Northern bobwhite exposed to 2A-DNT. Values represent the distribution of primary KEGG pathways enriched in differentially expressed transcripts or proteins that are sorted into 2^nd^ order KEGG ontology terms (listed in the legend). The vertical columns of legend terms track the charts clockwise starting at the twelve o’clock position. Specific pathways can be found in Additional file 1: Table S5

The most highly enriched pathways observed in liver tissues were those involved in lipid metabolism, carbohydrate metabolism and energy metabolism (Additional file 1: Table S5). Specifically, two pathways involved in lipid metabolism (fatty acid metabolism and steroid biosynthesis) were the most highly enriched pathways (p = 0.003 and 0.004, respectively). Investigation of transcript expression for specific pathways is provided in Additional file 1: Table S6 where, for example, decreased expression was observed for all significant transcripts involved in fatty acid metabolism (Fig. 2). Similarly, decreased expression was observed for all significant transcripts involved in steroid biosynthesis. The significantly enriched pathways involved in carbohydrate metabolism included pyruvate, glyoxylate and dicarboxylate metabolism (Fig. 2, Additional file 1: Table S5). Significantly enriched pathways having the most abundant representation in liver tissues of birds dosed with 2A-DNT were those involved in amino acid metabolism, carbohydrate metabolism, the endocrine system and energy metabolism in females whereas lipid metabolism, cell growth and death, carbohydrate metabolism, endocrine system, and energy metabolism were most abundant in males (Fig. 3, Additional file 1: Table S6). In addition to pathways involved in carbohydrate metabolism, the pentose and glucuronate interconversion pathway was significantly enriched. Enrichment of transcripts involved in multiple amino acid metabolic pathways was observed (Fig. 2) including tryptophan, phenylalanine, glycine, serine, threonine, cysteine, methionine metabolism, all of which occurred in females dosed at 3 mg/kg-d (Additional file 1: Table S5). Finally, pathways involved in endocrine system function were significantly enriched including peroxisome proliferator-activated receptor (PPAR) signaling (Fig. 2) and adipocytokine signaling pathway (Additional file 1: Table S5).

#### Kidney

No KEGG ontology categories or pathways were found in common among sexes in response to 2A-DNT dosing (Fig. 1h, Additional file 1: Table S5). The most highly enriched pathway for females was involved in signal molecules/interactions, specifically the extracellular matrix (ECM) receptor interaction. All transcripts had decreased expression within this pathway including six genes involved in collagen metabolism (NP_989757, NP_990121, NP_990438, NP_990679, NP_990865, XP_421847). In males, the most enriched pathways were involved in carbohydrate and amino acid metabolism (Additional file 1: Table S7). Butanoate metabolism, pyruvate metabolism and citrate cycle pathways involved in carbohydrate metabolism were each enriched at the 14 and 30 mg/kg-d 2A-DNT doses. Finally, the amino acid pathways enriched within males were primarily found at the lowest 2A-DNT dose. The most highly enriched pathway, valine, leucine and isoleucine biosynthesis was enriched at the 14 mg/kg-d dose.

### Proteomics

A total of 1547, 1586 and 1900 proteins were identified from male liver, female liver and male kidney tissues, respectively. Of these, 268, 245 and 224 proteins were differentially expressed in response to 2A-DNT in male liver, female liver and male kidney tissues, respectively (Additional file 1: Table S8). There was a higher degree of target-by-target commonality among sexes in the protein expression results (as high as 43 %) in contrast to that seen in the transcript expression data (Fig. 1b).

### Network analysis (Proteins)

#### Liver

The commonality of top gene networks affected in liver tissue among sexes ranged from 36-40 %. The top 4 most represented network functions affected in liver tissues of both males and females dosed with 2A-DNT included lipid metabolism, small molecule biochemistry, and nervous system development/function (Table 1).

#### Kidney

The top 5 network functions affected in male kidney tissue included endocrine system disorders, gastrointestinal disease, metabolic disease and cell-to-cell signaling and interaction (Additional file 1: Table S4).

### Canonical pathway (Proteins)

Significant protein enrichment was observed for 26 and 32 KEGG pathways in liver tissue of females and males, respectively, and 23 pathways in kidney of males, all in response to 30 mg/kg-d 2A-DNT (Additional file 1: Table S5). Commonality of KO terms enriched in liver tissue among males and females was 83-100 % (Fig. 1h).

#### Liver

The most highly enriched pathways observed in liver tissues were those involved in lipid metabolism, carbohydrate metabolism and amino acid metabolism for both male and female birds (Fig. 2, Additional file 1: Table S5). Fatty acid metabolism was the most highly enriched pathway for both males and females. Within this pathway, 14 proteins in females and 14 proteins in males had significant differential expression (Fig. 2) where 10 proteins were found in common among sexes (Additional file 1: Table S6). In males, 12 of 14 proteins had increased expression relative to controls whereas only 5 of 14 proteins had increased expression in females (Fig. 2). The glycolysis and gluconeogenesis pathway was the second most highly enriched pathway where 17 proteins were differentially expressed in both females and males (Fig. 2, Additional file 1: Table S5) with 11 in common among sexes (Additional file 1: Table S6). Both males and females exhibited predominantly increased protein expression within this pathway (12 of 17 in each sex, Fig. 2). A variety of pathways related to amino acid metabolism were enriched in response to 2A-DNT and are discussed in the Comparison of Proteomics and Transcriptomics Results section below.

#### Kidney

The most highly enriched pathways in kidney tissue of males exposed to 30 mg/kg-d doses of 2A-DNT were involved in signaling molecules/interaction, cell communication and carbohydrate metabolism, while the pathways having the greatest representation were involved in carbohydrate metabolism and amino acid metabolism (Additional file 1: Table S5). A member of the signaling molecules/interaction ontology, the ECM-receptor interaction pathway was the most highly enriched pathway wherein 6 proteins related to collagen metabolism were affected (Additional file 1: Table S7). The extracellular matrix (ECM) receptor interaction pathway represented a subset of another enriched pathway involved in cell communication, the focal adhesion pathway (Additional file 1: Table S5). Finally, the glycolysis/gluconeogenesis pathway had the greatest enrichment among pathways involved in carbohydrate metabolism where 9 out of 12 proteins had decreased expression (Additional file 1: Table S7).

### Comparison of proteomics and transcriptomics results

Direct protein-to-transcript comparisons of targets differentially expressed in response to the 30 mg/kg-d dose of 2A-DNT indicated that only 2.4, 3.7 and 3.7 % of all differentially expressed protein and transcript targets were similar in female liver, male liver and male kidney tissues, respectively (Fig. 1c) and correlations between fold change values of these targets were weak (Additional file 2: Figure S1). In contrast, network functions enriched among protein and transcript results indicated a greater degree of commonality (42, 36 and 27 % for female liver, male liver and male kidney tissues, respectively) in the 30 mg/kg-d dose treatment (Fig. 1f). Similarly, KO terms enriched among protein and transcript results were closely related with as much as 83, 66 and 100 % correspondence for female liver, male liver and male kidney tissues, respectively in the 30 mg/kg-d dose treatment (Fig. 1i, Fig. 3).

### Liver

In liver tissues of females, network functions found in common among protein and transcript results included: lipid metabolism, small molecule biochemistry, cell growth/proliferation, cell death, and nervous system development/function. For males, lipid metabolism, small molecule biochemistry, molecular transport and cellular development were found in common in liver tissue. The topology for two of the top 5 networks representing “lipid metabolism, molecular transport, small molecule biochemistry” indicated shared motifs and network hubs among transcript and protein expression results (Additional file 2: Figure S2). Canonical pathways enriched in response to 2A-DNT dosing for both transcriptomic and proteomic results included those involved in lipid metabolism, carbohydrate metabolism, amino acid metabolism, energy metabolism, endocrine system, and translation (Additional file 1: Table S5). The first three of these pathway categories were both highly abundant and highly enriched. Lipid metabolism had the most strongly enriched pathways for both protein and transcript expression data, wherein fatty acid metabolism was a predominantly affected pathway (Fig. 2). Carbohydrate metabolism was the next most enriched pathway category within which pyruvate metabolism, glyoxylate/dicarboxylate metabolism and pentose/glucuronate interconversions were affected in common. Pyruvate metabolism was the most enriched pathway across transcript and protein results where malic enzyme 1, NADP (+)-dependent, cytosolic (NP_989634), lactate dehydrogenase A (NP_990615) and acetyl-Coenzyme A carboxylase alpha (NP_990836) were found in common among expression results although expression levels were mixed across sexes and 2A-DNT doses (Additional file 1: Table S6). A variety of significantly enriched pathways involved in amino acid metabolism were found in common among transcript and protein expression data including tryptophan, phenlylalanine, glycine, serine, threonine, cysteine and methionine metabolism (Table S5). Predominantly increased expression was observed for these pathways, particularly in males (Additional file 1: Table S6). Finally, the endocrine-related PPAR signaling pathway, which serves as a primary regulator of lipid metabolism [14], was significantly enriched in both protein and transcript expression results (Additional file 1: Tables S5 and S6).

### Kidney

In kidney tissues, network functions found in common among transcriptomic and proteomic results included: cell-to-cell signaling/interaction, cellular assembly/organization, and tissue development (Additional file 1: Table S4). Canonical pathways enriched in common among transcriptomic and proteomic results sets included those involved in signaling molecules/interactions, carbohydrate metabolism, amino acid metabolism, lipid metabolism, endocrine system, and cell communication (Additional file 1: Table S5). A member of the signaling molecules/interactions pathway, the ECM-receptor interaction, was the most highly enriched pathway for both protein and transcript results sets. Interestingly, this enrichment was observed in females for all 2A-DNT doses but not in male transcriptomics results, however this enrichment was observed in the male proteomics results. A similar finding was observed when investigating the highly related focal adhesion pathway that is involved in cell communication (Additional file 1: Table S5). Across these pathways, decreased expression of collagen, type VI, alpha 1, 2 and 3 (NP_990438, NP_990679, NP_990865) was observed in both transcript and protein expression results (Additional file 1: Table S7). However, protein expression for specific elements that compose renal basement membranes [27, 28] including: collagen IVα1 (NP_990438) and IVα2 (NP_001155862); laminin β1 (XP_415943), β2 (NP_989497), and γ2 (XP_422285); and heparan sulfate proteoglycan 2 (XP_427362, NP_001001876) was increased in male kidneys (Additional file 1: Table S7). Within carbohydrate metabolism, the butanoate, citrate cycle, and pyruvate metabolism pathways were enriched in common among transcript and protein results. Within pathways related to the endocrine system, PPAR signaling was enriched where both protein and transcript expression was predominantly decreased (Additional file 1: Table S7). Overall, a number of pathway-level responses to 2A-DNT were conserved among transcript and protein expression in Northern bobwhite kidney tissue.

#### PPAR nuclear activation bioassays

**Fig. 4:**
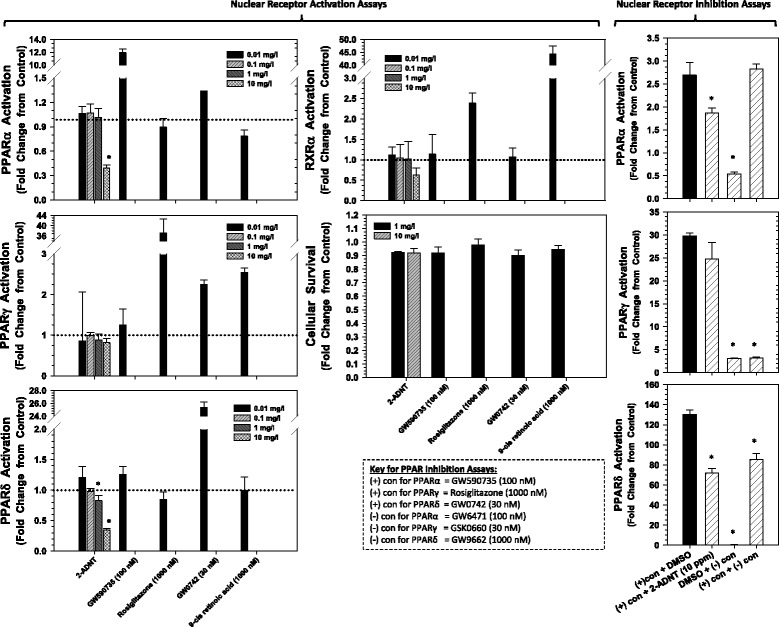
Effects of 2A-DNT in nuclear receptor activation and inhibition assays conducted in Chinese hamster ovary cells. The effects of 2A-DNT were examined against positive and negative controls for each nuclear receptor. Asterisks represent significant differences among treatments

2A-DNT caused a significant decrease in PPAR nuclear signaling in nuclear receptor activation and inhibition assays (Fig. 4). Significant reduction in signaling for PPARα was observed at 10 mg/L and at 1 and 10 mg/L for PPARδ (Fig. 4). Conversely, 2A-DNT had no significant effects on PPARγ and RXRα signaling. The significant reductions in PPARα and PPARδ signaling in the nuclear receptor inhibition assays indicate that 2A-DNT can effectively compete against the native ligand for binding to each respective receptor. Finally, neither exposure to 2A-DNT or positive controls affected cellular survival (Fig. 4).

## Discussion

We utilized comparative and integrative approaches for interpreting transcriptomics and proteomics results into hypothetical mechanisms of action underlying observed effects of 2A-DNT exposure in Northern bobwhite. A brief summary of the subchronic effects of 2A-DNT described in Quinn et al. [6] includes: mortality in the 14 and 30 mg/kg-d doses in females (none in males), significantly increased liver:brain weight ratios in each sex at 30 mg/kg-d, significantly increased plasma triglycerides in males at 0.5 and 3 mg/kg-d, significantly decreased leukocyte counts in females at 30 mg/kg-d, and splenic reticular cell hyperplasia in one male and two females at 30 mg/kg-d. Additionally, behavioral changes were observed including increased feeding in males at 0.5, 3 and 14 mg/kg-d at days 39–60 and increased feeding in females at the same dose levels from days 4–25. Although feeding levels differed across 2A-DNT doses, no significant changes in body weight or growth were observed [6]. Given the long-term subchronic (60d) exposure period in adult animals, the experimental design allowed observation of transcript and protein expression in response to 2A-DNT where gene expression signaling had the opportunity to approach a relatively steady-state condition [29]. The similarity of effects at the transcriptional and protein levels were considerably greater at higher levels of organization such as the top enriched biological networks (Fig. 1f) and enriched KEGG canonical metabolic pathways (Fig. 3) compared to direct transcript to protein comparisons (Fig. 1c). Given that the KEGG metabolic pathways and IPA-curated biological networks represent integrated systems of genes/processes that execute higher-order biological functions [17, 19], enrichment of differentially expressed genes within these pathways/networks were more readily translated to apical phenotypes.

### Matching pathways/Networks to phenotypes (Mechanisms of Action)

#### Mortality

All males survived the subchronic 60d exposure to 2A-DNT however, 4 females were either found dead or were moribund and euthanized, three in the 14 mg/kg-d treatment and one in the 30 mg/kg-d treatment [6]. Necropsy of these females revealed effects including: edematous gastrointestinal tracts, green food contents in gizzards, pale kidneys, enlarged gall bladders, regressed ovaries and apparent necrosis in the liver [6]. Additionally, moribund animals were unable to stand and experienced tremors during handling. Many of the same effects were also observed in Northern bobwhite exposed to a structurally-related nitroaromatic, 2,6-dinitrotoluene [9]. Mortality represents a complex toxicological endpoint that generally results from the failure of a single or multiple critical life-support systems. Protein and transcript expression were examined in animals that survived the 2A-DNT treatments, however we focused on phenotypes characteristic of those found in the quail that were lethally affected. These phenotypes represent likely indicators of system failures that contributed to animal death.

Expression in female liver tissue from the 14 and 30 mg/kg-d dosing treatments for the transcriptomic data and at the 30 mg/kg-d dosing in the proteomic data revealed enrichment of the IPA network “cell death” (Table 1) which is likely indicative of observed necrosis in liver. Within differentially expressed transcripts, the IPA sub-network “liver necrosis/cell death” was significantly enriched at both 14 and 30 mg/kg-d dosed females. Differentially expressed genes within this sub-network included: caspase 3 (CASP3, NP_990056, −2.6 fold change at 30 mg/kg-d), Insulin-like growth factor 1 (IGF1, NP_001004384, 2.17 fold change at 30 mg/kg-d) and cyclin-dependent kinase inhibitor 1B (CDKN1B, NP_989587, 1.62 fold change at 14 mg/kg-d), (Additional file 1: Table S2). CASP3 represents a cysteinyl-protease that is an initiator of programmed cell death [30], while IGF1 acts as an inhibitor of programmed cell death [31] and CDKN1B is a cyclin-dependent kinase inhibitor which arrests cells in the G1 phase of the cell cycle [32]. The combination of decreased expression of CASP3 and increased expression of CDKN1B is suggestive of a molecular-level response for preserving liver cell integrity, and reduced potential for cellular multiplication through decreased expression of CDKN1B. In parallel with transcript expression results, differential expression of proteins in female liver represented within the “cell death” network was also observed including: MYC induced nuclear antigen (MINA, XP_423066, −1.70 fold change at 30 mg/kg-d) and prohibitin 2 (PHB2, NP_001074354, −1.03 fold change at 30 mg/kg-d), (Additional file 1: Table S8). Both MINA and PHB2 are involved in increasing cell proliferation [33, 34] and observed decreases in expression of each is further indication of a molecular response to arrest cellular multiplication. Taken in total, both the transcript and protein expression results indicated molecular responses consistent with preservation of liver cells and inhibition of cellular multiplication in response to 2A-DNT dosing.

Integrated impacts on higher order biological systems and processes also likely contributed to lethality. For example, effects on PPAR metabolism, lipid metabolism, carbohydrate metabolism and amino acid metabolism likely represent interferences with systemic energy budgets as we have observed in other nitrotoluenes [11, 13, 15]. These contributing factors are described in more detail below.

#### Increased liver weights: connections to xenobiotic and energy metabolism

A variety of metabolic functions that primarily occur in the liver had enriched expression in response to 2A-DNT dosing. Enrichment of xenobiotic metabolism and multiple pathways involved in energy metabolism provide indicators of the potential causes of increased liver weights as well as systemic impacts of nitrotoluene exposure.

### Xenobiotic metabolism

The proteomics results indicated impacts on the “drug metabolism” gene network and the “xenobiotic biodegradation and metabolism” pathway. Significantly increased expression of a protein similar to cytochrome P450 2D20 was observed in both males and females (XP_416219) whereas cytochrome P450 CYP3A37 (NP_001001751) and CYP1A1 (NP_990477) protein expression in liver was significantly reduced in females (Additional file 1: Table S6). A variety of P450 transcripts were similarly differentially expressed, although were not statistically enriched within a canonical pathway (Additional file 1: Table S2). As a class of enzymes P450’s are known to participate in phase I metabolism of endogenous and xenobiotic compounds [35]. Although initiated as a detoxification mechanism, metabolism of certain xenobiotics by P450s can result in highly toxic metabolite formation, as has been observed in certain CYP1A1-catalyzed reactions [36]. Such potentiation events may be the cause of the reduced expression of CYP1A1 and possibly CYP3A37 although the response could also be related to endogenous metabolism specific to females. There was mixed protein expression for glutathione transferase in both males and females, however the cytosolic classes of the gene had increased expression including glutathione transferase zeta 1 (XP_00123362 and XP_001233653) in both sexes. Although not significantly enriched as a pathway, transcripts for a variety of glutathione transferases were also differentially expressed in both males and females, all with increased expression (Additional file 1: Table S2). Glutathione transferases execute phase II xenobiotic metabolism where glutathione is conjugated to the xenobiotic to increase solubility for potential elimination [35]. Overall, protein expression in liver tissue for both males and females indicated increased expression for elements of both phase I and II xenobiotic metabolism in response to 2A-DNT dosing indicating the potential for enhanced metabolic activity for 2A-DNT detoxification in the liver. As a consequence of the increased need for xenobiotic metabolism, hepatomegaly may have occurred [37] to provide increased metabolic capacity for the degradation and excretion of 2A-DNT. Finally, the unique responses of females in the expression of transcripts & proteins involved in programmed cell death, arrest of cell cycle and various cytochrome P450s provides potential sources for the observations of increased sensitivity of females to 2A-DNT dosing relative to males.

### Lipid metabolism

An additional putative cause for the observed increase in liver weights resulting from 2A-DNT dosing is impaired potential for lipid metabolism. Wintz et al. [38] observed this effect in fathead minnow exposed to a nitrotoluene closely-related to 2A-DNT, 2,4-dinitrotoluene, where liver weights were increased due to increased lipid content that was attributed to impaired lipid catabolic pathways. Nitrotoluenes have been observed to impact transcriptional expression of genes involved in lipid metabolism [11, 13, 15, 38, 39] as well as downstream lipid content [13, 38, 39]. In the current study, enrichment of lipid metabolism pathways in response to 2A-DNT dosing were principle findings within both gene networks and canonical pathways for both transcriptomic and proteomics expression in liver tissue (Table 1, Figs. 1 and 2, Additional file 1: Tables S4 and S5). This very robust response was observed in both sexes, and given the similarities in findings for other nitrotoluenes described above, our study provides further evidence that impacts on lipid metabolism are a principle affect of nitrotoluenes in a variety of species. Although manifested through potential increases in liver weight, the negative impact of nitrotoluenes on lipid metabolism has the potential for systemic impacts on energy metabolism that may negatively impact overall individual health and performance of physical activity as described by Wilbanks et al. [15].

### Carbohydrate pathways

Both gene networks and canonical pathways involved in carbohydrate metabolism were enriched in transcript and protein expression datasets in liver tissue. (Table 1, Figs. 1 and 2, Additional file 1: Tables S4 and S5). As with lipid metabolism, nitrotoluenes have been observed to affect transcription of genes involved in carbohydrate metabolism and related pathways involved in energy metabolism [11, 13, 15]. Wilbanks et al. [15] indicated that the increases in carbohydrate metabolism occurred in 2,4-DNT exposures as a likely compensatory mechanism to maintain energy budgets given the impaired potential to utilize lipid as an energy substrate.

### PPAR (transcriptional regulator of both lipid and carbohydrate pathways)

**Fig. 5:**
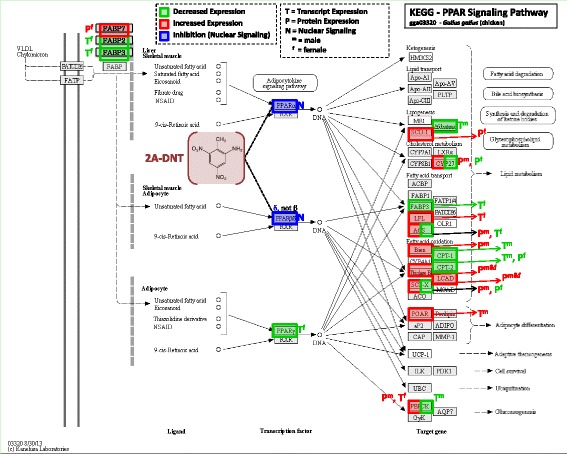
Effects of 2A-DNT on transcript expression, protein expression and nuclear signaling mapped onto the Kyoto Encyclopedia of Genes and Genomes (KEGG) peroxisome proliferator-activated receptor (PPAR) pathway

PPAR signaling pathways represent principle regulators of lipid metabolism [14, 40] and carbohydrate metabolism [14, 41]. PPAR signaling pathways were significantly enriched in response to 2A-DNT in liver tissue for both transcript and protein expression datasets (Fig. 5, Additional file 1: Table S5). Similar responses have been observed for multiple nitrotoluenes (i.e. TNT, 2,4,-DNT, and 2,6-DNT) ranging across a number of species including: Northern bobwhite, rat, mouse, fathead minnow and *Daphnia magna* [11, 13, 15, 38, 39]. All of the studies have implicated nitrotoluene antagonism of PPAR signaling as a potential molecular initiating event (MIE) for impaired lipid metabolism and energy budgets.

*In vitro* nuclear-signaling bioassays indicated that 2A-DNT is an antagonist of human PPARα and PPARδ nuclear signaling (Fig. 4). Given that the amino acid sequence for *Gallus gallus* PPARα and PPARδ are 89 % and 90 % identical to their human orthologs (http://blast.ncbi.nlm.nih.gov), respectively, a similar response to 2A-DNT is plausible in Northern bobwhite and is consistent with observed effects on lipid metabolic pathways (Fig. 5, Additional file 1: Table S6). The nitrotoluene 2,4-DNT has also been found to antagonize PPARα using human *in vitro* nuclear-activation assays [15]. Further, the 2,4-DNT induced antagonism of PPARα signaling acted as the principal MIE for impaired energy metabolism that led to adverse outcomes of reduced body weights and diminished exercise performance in mice. Examination of 2A-DNT effects (Fig. 5) suggests this response may be conserved in Northern bobwhite with at least some similar down-stream impacts on molecular pathways involved in energy metabolism including both lipid metabolism and gluconeogenesis (a key carbohydrate metabolic pathway). Curiously, although transcriptomics results indicated predominantly decreased expression of genes involved in fatty acid metabolism, certain proteins involved in this process were over-expressed (Fig. 5, Additional file 1: Table S6). In spite of this increased expression, plasma-triglyceride levels were not affected in females and were actually increased in males, although this may have been influenced by increased feeding rates in males [6]. 2A-DNT impacts PPAR nuclear signaling and expression of genes controlling lipid and carbohydrate metabolism, as observed with other nitrotoluenes [11, 15], however, 2A-DNT did not cause decreased body weight suggesting a decreased relative potency of 2A-DNT in comparison to other nitrotoluenes (i.e. TNT and 2,4-DNT) with respect to effects on energy metabolism.

#### Decreased leukocyte counts

Leukocyte counts were decreased in females at the highest 2A-DNT dose [6]. Neither the metabolic pathway nor the gene-network analyses provided strong indications of potential causes for this observation given transcriptomic or proteomic data. However, significantly decreased protein expression for catalase (CAT, XP_001233111) was observed in females dosed at 30 mg/kg-d (Additional file 1: Table S8). Catalase maintains redox potential under oxidative stress allowing the immune system to sustain stable function [42]. Additionally, decreased transcriptional expression of B-cell CLL/lymphoma 6 (zinc finger protein 51), a transcriptional repressor of genes involved in B-cell maturation (http://www.uniprot.org/uniprot/P41182), was observed in females at both the 14 and 30 mg/kg-d doses. Although, not enriched in pathways or gene networks, both the transcriptomics and proteomics results do provide indicators of potential mechanisms leading to decreased leukocyte counts in females.

#### Extracellular matrix and focal adhesion expression in kidney

Although the only apical evidence of effects on 2A-DNT dosing in Northern bobwhite kidneys was pale coloration, both transcriptomic and proteomic results indicated enrichment of extracellular matrix (ECM) receptor interactions and focal adhesion pathways. Predominant effects within both pathways were altered expression in collagen and collagen precursors along with effects on laminin and additional proteins that interact with collagen (Additional file 1: Table S7). Interactions between collagen IV and laminin are known to be important for guiding the structural development of the kidney [28] and, in combination with heparan sulfate proteoglycans are primary components of the renal basement membrane [27, 28]. Protein expression for each of these elements including: collagen IVα1 (NP_990438) and IVα2 (NP_001155862); laminin β1 (XP_415943), β2 (NP_989497), and γ2 (XP_422285); and heparan sulfate proteoglycan 2 (XP_427362, NP_001001876) was increased in male kidneys (Additional file 1: Table S7) possibly in order to maintain the integrity of basement membranes within the kidney that are key to renal function [27]. Both transcript and protein expression for various other collagen types that are not involved with renal basement membrane structure were decreased potentially as a trade off for mobilizing increased collagen IV. However, additional research investigating specific kidney histochemistry and physiology in response to 2A-DNT dosing is needed to validate such hypotheses as presented herein.

## Conclusions

This work compared and integrated transcript and protein expression in response to an important environmental pollutant in a sensitive avian model species providing a systems toxicology evaluation of adverse-exposure effects. The direct correspondence between transcript and protein expression in response to 2A-DNT was poor (Table 1), which is not unexpected [43]. Much greater similarity was observed in higher order functional responses including enrichment of molecular networks and canonical metabolic pathways (Table 1, Fig. 3), especially for molecular functions underlying observed toxicological phenotypes. For example , both transcript and protein expression results indicated molecular responses consistent with inhibition of programmed cell death and arrest of cell cycle in liver tissues of females at doses of 2A-DNT that caused liver necrosis and death in females. Additionally, both transcript and protein expression in liver tissue was indicative of induced phase I and II xenobiotic metabolism as a likely response to detoxify and excrete 2A-DNT. Nuclear signaling, transcript expression and protein expression assays each implicated PPAR nuclear signaling as a primary molecular target in the 2A-DNT exposure with significant downstream enrichment of PPAR-regulated pathways including various lipid metabolic pathways and gluconeogenesis within carbohydrate pathways in liver tissue. The relative expression of transcripts and proteins within enriched pathways was at times divergent indicating dynamics in expression that would likely be better characterized in a time series exposure relative to a dose series. None the less, the transcript expression assays identified many critical metabolic pathways as well as a key molecular initiating event known to be affected in nitrotoluene exposures identified in protein and nuclear signaling assays, respectively. Although the differential expression of transcripts and proteins was largely unique, the consensus of functional pathways and gene networks enriched among transcriptomic and proteomic datasets provided the identification of many critical metabolic functions underlying 2A-DNT toxicity as well as impaired PPAR signaling, a key molecular initiating event known to be affected in di- and trinitrotoluene exposures.

## Additional files

Additional file 1:
**Supplemental tables.** (XLSX 1436 kb) Additional file 2:
**Supplemental text and figures.** (DOCX 847 kb)

## References

[1] Jenkins TFP JC, Ranney TA, Berry TE, Miyares PH, Walsh ME, Hewitt AD, Perron NM, Parker LV, Hayes CA, Wahlgren E (2001). Characterization of explosives contamination at military firing ranges. vol. ERDC/CRREL TR-01-5.

[2] Talmage SS, Opresko DM, Maxwell CJ, Welsh CJ, Cretella FM, Reno PH, Daniel FB (1999). Nitroaromatic munition compounds: environmental effects and screening values. Rev Environ Contam Toxicol.

[3] Esteve-Nunez A, Caballero A, Ramos JL (2001). Biological degradation of 2,4,6-trinitrotoluene. Microbiol Mol Biol Rev.

[4] Hovatter PAT, S. S.; Opresko, D. M.; Ross, R. H.: Ecotoxicity of Nitroaromatics to Aquatic and Terrestrial Species at Army Superfund Sites. In: Environmental Toxicology and Risk Assessment: Modelinq and Risk Assessment Edited by Dwyer FJD, T.R.; Hinman, M.L., vol. 6th. American Society for Testing and Materials; West Conshohocken, PA. 1997.

[5] Gionfriddo JP, Best LB (1996). Grit-Use patterns in north american birds: the influence of diet, body size, and gender. The Wilson Bulletin.

[6] Quinn MJ, McFarland CA, Lafiandra EM, Bazar MA, Johnson MS (2010). Acute, subacute, and subchronic exposure to 2A-DNT (2-amino-4,6-dinitrotoluene) in the northern bobwhite (Colinus virginianus). Ecotoxicology.

[7] Quinn MJ, Hanna TL, Shiflett AA, McFarland CA, Cook ME, Johnson MS, Gust KA, Perkins EJ (2013). Interspecific effects of 4A-DNT (4-amino-2,6-dinitrotoluene) and RDX (1,3,5-trinitro-1,3,5-triazine) in Japanese quail, Northern bobwhite, and Zebra finch. Ecotoxicology.

[8] Johnson MS, Quinn MJ, Bazar MA, Gust KA, Escalon BL, Perkins EJ (2007). Subacute toxicity of oral 2,6-dinitrotoluene and 1,3,5-trinitro-1,3,5-triazine (RDX) exposure to the northern bobwhite (Colinus virginianus). Environ Toxicol Chem.

[9] Quinn MJ, Bazar MA, McFarland CA, Perkins EJ, Gust KA, Gogal RM, Johnson MS (2007). Effects of subchronic exposure to 2,6-dinitrotoluene in the northern bobwhite (Colinus virginianus). Environ Toxicol Chem.

[10] Gust KA, Pirooznia M, Quinn MJ, Johnson MS, Escalon L, Indest KJ, Guan X, Clarke J, Deng Y, Gong P (2009). Neurotoxicogenomic investigations to assess mechanisms of action of the munitions constituents RDX and 2,6-DNT in Northern bobwhite (Colinus virginianus). Toxicol Sci.

[11] Rawat A, Gust KA, Deng Y, Garcia-Reyero N, Quinn MJ, Johnson MS, Indest KJ, Elasri MO, Perkins EJ (2010). From raw materials to validated system: the construction of a genomic library and microarray to interpret systemic perturbations in Northern bobwhite. Physiol Genomics.

[12] Rawat A, Gust KA, Elasri MO, Perkins EJ (2010). Quail Genomics: a knowledgebase for Northern bobwhite. BMC Bioinformatics.

[13] Deng Y, Meyer SA, Guan X, Escalon BL, Ai J, Wilbanks MS, Welti R, Garcia-Reyero N, Perkins EJ (2011). Analysis of common and specific mechanisms of liver function affected by nitrotoluene compounds. PLoS One.

[14] Desvergne B, Wahli W (1999). Peroxisome proliferator-activated receptors: nuclear control of metabolism. Endocr Rev.

[15] Wilbanks MS, Gust KA, Atwa S, Sunesara I, Johnson D, Ang CY, Meyer SA, Perkins EJ (2014). Validation of a genomics-based hypothetical adverse outcome pathway: 2,4-dinitrotoluene perturbs PPAR signaling thus impairing energy metabolism and exercise endurance. Toxicol Sci.

[16] Calvano SE, Xiao W, Richards DR, Felciano RM, Baker HV, Cho RJ, Chen RO, Brownstein BH, Cobb JP, Tschoeke SK (2005). A network-based analysis of systemic inflammation in humans. Nature.

[17] Viswanathan GA, Seto J, Patil S, Nudelman G, Sealfon SC (2008). Getting started in biological pathway construction and analysis. PLoS Comput Biol.

[18] Dennis G, Sherman BT, Hosack DA, Yang J, Gao W, Lane HC, Lempicki RA (2003). DAVID: database for annotation, visualization, and integrated discovery. Genome Biol.

[19] Curtis RK, Oresic M, Vidal-Puig A (2005). Pathways to the analysis of microarray data. Trends Biotechnol.

[20] Ankley GT, Bennett RS, Erickson RJ, Hoff DJ, Hornung MW, Johnson RD, Mount DR, Nichols JW, Russom CL, Schmieder PK (2010). Adverse outcome pathways: a conceptual framework to support ecotoxicology research and risk assessment. Environ Toxicol Chem.

[21] Baldi P, Long AD (2001). A Bayesian framework for the analysis of microarray expression data: regularized t -test and statistical inferences of gene changes. Bioinformatics.

[22] Rawat A, Seifert GJ, Deng Y (2008). Novel implementation of conditional co-regulation by graph theory to derive co-expressed genes from microarray data. BMC Bioinformatics.

[23] van den Berg BH, Harris T, McCarthy FM, Lamont SJ, Burgess SC (2007). Non-electrophoretic differential detergent fractionation proteomics using frozen whole organs. Rapid Commun Mass Spectrom.

[24] Vergnon JM, Barthelemy JC, Riffat J, Boissier C, Claudy A, Emonot A (1992). Raynaud's phenomenon of the lung. A reality both in primary and secondary Raynaud syndrome. Chest.

[25] Yates JR, Eng JK, McCormack AL, Schieltz D (1995). Method to correlate tandem mass spectra of modified peptides to amino acid sequences in the protein database. Anal Chem.

[26] Pendarvis K, Kumar R, Burgess SC, Nanduri B (2009). An automated proteomic data analysis workflow for mass spectrometry. BMC Bioinformatics.

[27] Miner JH (1999). Renal basement membrane components. Kidney Int.

[28] Laurie GW, Horikoshi S, Killen PD, Segui-Real B, Yamada Y (1989). In situ hybridization reveals temporal and spatial changes in cellular expression of mRNA for a laminin receptor, laminin, and basement membrane (type IV) collagen in the developing kidney. J Cell Biol.

[29] Legewie S, Herzel H, Westerhoff HV, Bluthgen N (2008). Recurrent design patterns in the feedback regulation of the mammalian signalling network. Mol Syst Biol.

[30] Ghavami S, Hashemi M, Ande SR, Yeganeh B, Xiao W, Eshraghi M, Bus CJ, Kadkhoda K, Wiechec E, Halayko AJ (2009). Apoptosis and cancer: mutations within caspase genes. J Med Genet.

[31] Chattopadhyay A, Carpenter G (2002). PLC-gamma1 is required for IGF-I protection from cell death induced by loss of extracellular matrix adhesion. J Cell Sci.

[32] Polyak K, Lee MH, Erdjument-Bromage H, Koff A, Roberts JM, Tempst P, Massague J (1994). Cloning of p27Kip1, a cyclin-dependent kinase inhibitor and a potential mediator of extracellular antimitogenic signals. Cell.

[33] Tsuneoka M, Koda Y, Soejima M, Teye K, Kimura H (2002). A novel myc target gene, mina53, that is involved in cell proliferation. J Biol Chem.

[34] Merkwirth C, Dargazanli S, Tatsuta T, Geimer S, Lower B, Wunderlich FT, von Kleist-Retzow JC, Waisman A, Westermann B, Langer T (2008). Prohibitins control cell proliferation and apoptosis by regulating OPA1-dependent cristae morphogenesis in mitochondria. Genes Dev.

[35] Parkinson A, CD K (2001). Biotransformation of Xenobiotics. Casarett and Doull’s Toxicology: The Basic Science of Poisons.

[36] Gilday D, Gannon M, Yutzey K, Bader D, Rifkind AB (1996). Molecular cloning and expression of Two novel avian cytochrome P450 1A enzymes induced by 2,3,7,8-tetrachlorodibenzo-p-dioxin. J Biol Chem.

[37] Huang W, Zhang J, Washington M, Liu J, Parant JM, Lozano G, Moore DD (2005). Xenobiotic stress induces hepatomegaly and liver tumors via the nuclear receptor constitutive androstane receptor. Mol Endocrinol.

[38] Wintz H, Yoo LJ, Loguinov A, Wu YY, Steevens JA, Holland RD, Beger RD, Perkins EJ, Hughes O, Vulpe CD (2006). Gene expression profiles in fathead minnow exposed to 2,4-DNT: correlation with toxicity in mammals. Toxicol Sci.

[39] Stanley JK, Perkins EJ, Habib T, Sims JG, Chappell P, Escalon BL, Wilbanks M, Garcia-Reyero N (2013). The good, the bad, and the toxic: approaching hormesis in Daphnia magna exposed to an energetic compound. Environ Sci Technol.

[40] Wolfrum C, Borrmann CM, Borchers T, Spener F (2001). Fatty acids and hypolipidemic drugs regulate peroxisome proliferator-activated receptors alpha - and gamma-mediated gene expression via liver fatty acid binding protein: a signaling path to the nucleus. Proc Natl Acad Sci U S A.

[41] Millward CA, Desantis D, Hsieh CW, Heaney JD, Pisano S, Olswang Y, Reshef L, Beidelschies M, Puchowicz M, Croniger CM (2010). Phosphoenolpyruvate carboxykinase (Pck1) helps regulate the triglyceride/fatty acid cycle and development of insulin resistance in mice. J Lipid Res.

[42] Wang C, Yue X, Lu X, Liu B (2013). The role of catalase in the immune response to oxidative stress and pathogen challenge in the clam Meretrix meretrix. Fish Shellfish Immunol.

[43] Guo Y, Xiao P, Lei S, Deng F, Xiao GG, Liu Y, Chen X, Li L, Wu S, Chen Y (2008). How is mRNA expression predictive for protein expression? A correlation study on human circulating monocytes. Acta Biochim Biophys Sin.

